# Clock Synchronization in Wireless Sensor Networks: An Overview

**DOI:** 10.3390/s90100056

**Published:** 2009-01-06

**Authors:** Ill-Keun Rhee, Jaehan Lee, Jangsub Kim, Erchin Serpedin, Yik-Chung Wu

**Affiliations:** 1 Department of Electronic Engineering, Hannam University / 133 Ojung-Dong, Daeduck-Gu, Daejun, 306-791, Korea; E-Mail: ikrhee@hnu.kr; 2 Department of Electrical and Computer Engineering, Texas A&M University / TAMU 3128, College Station, TX 77840-3128, USA; E-Mails: jhlee518@tamu.edu; jangsub@ece.skku.ac.kr; 3 Department of Electrical and Electronic Engineering, The University of Hong Kong, Hong Kong; E-Mail: ycwu@eee.hku.hk

**Keywords:** Wireless sensor networks, Clock synchronization, Clock offset, Clock skew

## Abstract

The development of tiny, low-cost, low-power and multifunctional sensor nodes equipped with sensing, data processing, and communicating components, have been made possible by the recent advances in micro-electro-mechanical systems (MEMS) technology. Wireless sensor networks (WSNs) assume a collection of such tiny sensing devices connected wirelessly and which are used to observe and monitor a variety of phenomena in the real physical world. Many applications based on these WSNs assume local clocks at each sensor node that need to be synchronized to a common notion of time. This paper reviews the existing clock synchronization protocols for WSNs and the methods of estimating clock offset and clock skew in the most representative clock synchronization protocols for WSNs.

## 1. Introduction

The recent advances in micro-electro-mechanical systems technology have expedited the development of tiny, low-cost, low-power, and multifunctional sensing devices, which are capable of performing tasks such as sensing, data processing, and communication [1-4]. A wireless sensor network (WSN) is a distributed network consisting, in general, of a large number of sensor nodes, which are densely deployed over a wide geographical region to track a certain physical phenomenon. The positions of wireless sensor nodes need not be engineered or predetermined. This enables random deployment in inaccessible terrains or during disaster relief operations. Therefore, this implies a need for wireless sensor network protocols and algorithms with self-organizing capabilities. Another unique feature of wireless sensor networks is the collaborative effort of sensor nodes to perform tasks such as data fusion, detection and measurement. Instead of sending the raw data to the destination node, sensor nodes use their own processing abilities to locally perform simple computations and transmit only the required and partially processed data. In other words, data from each sensor is collected to produce a single meaningful result value [5].

Wireless sensor networks can be applied to a wide range of applications in domains as diverse as medical [10], industrial, military [6], environmental [7-9], scientific [11-16], and home networks [10, 17-20]. Specifically, WSNs enable doctors to identify predefined symptoms by monitoring the physiological data of patients remotely. As a military application, WSNs can be used to detect nuclear, biological, and chemical attacks and presence of hazardous materials, prevent enemy attacks by means of alerts when enemy aircrafts are spotted, and monitor friendly forces, equipment and ammunition. Moreover, WSNs are also conducive to monitoring forest fire, observing ecological and biological habitats, and detecting floods and earthquakes. In terms of civilian applications of WSNs, it is possible to determine spot availability in a public parking lot, track active badge at the workplace, observe security in public places such as banks and shopping malls, and monitor highway traffic in a certain time. Additionally, WSNs can meet the needs for scientific applications such as space and interplanetary exploration, high energy physics, and deep undersea exploration [21].

Since the sensors in a wireless sensor network operate independently, their local clocks may not be synchronized with one another. This can cause difficulties when trying to integrate and interpret information sensed at different nodes. For instance, if a moving car is detected at two different times along a road, before we can even tell in what direction the car is going, the detection times have to be compared meaningfully. In addition, we must be able to transform the two time readings into a common frame of reference before estimating the speed of the vehicle. Estimating time differences across nodes accurately is also important in node localization. For example, many localization algorithms use ranging technologies to estimate internode distances; in these technologies, synchronization is needed for time-of-flight measurements that are then transformed into distances by multiplying with the medium propagation speed for the type of signal used such as radio frequency or ultrasonic. There are additional examples where cooperative sensing requires the nodes involved to agree on a common time frame such as configuring a beam-forming array and setting a TDMA (Time Division Multiple Access) radio schedule [22]. These situations mandate the necessity of one common notion of time in wireless sensor networks. Therefore, currently there is a huge interest towards developing energy efficient clock synchronization protocols to provide a common notion of time.

The clock synchronization problem has been studied thoroughly in the areas of Internet and local-area networks (LANs) for the last several decades. Many existing synchronization algorithms rely on the clock information from GPS (Global Positioning System). However, GPS-based clock acquisition schemes exhibit some weaknesses: GPS is not ubiquitously available (for example, underwater, indoors, under foliage) and requires a relatively high-power receiver, which is not possible in tiny and cheap sensor nodes. This is the motivation for developing software-based approaches to achieve in-network time synchronization. Among many protocols that have been devised for maintaining synchronization in Computer Networks, NTP (Network Time Protocol) [23] is outstanding owing to its ubiquitous deployment, scalability, robustness related to failures, and self-configuration in large multihop networks. Moreover, the combination of NTP and GPS has shown that it is able to achieve high accuracy on the order of a few microseconds [24]. However, NTP is not suitable for a wireless sensor environment, since wireless sensor networks pose numerous challenges of their own; to name a few, limited energy and bandwidth, limited hardware, latency, and unstable network conditions caused by mobility of sensors, dynamic topology, and multi-hopping. Hence, clock synchronization protocols different from the conventional protocols are needed in order to deal with the challenges specific to WSNs.

The aim of this paper is twofold. First, it surveys and reviews clock synchronization protocols mainly used in wireless sensor networks. Next, this paper describes a few methods for estimating clock offsets and skews in several representative clock synchronization schemes, and finally some theoretical results as well as simulation results are presented to illustrate the performances of different estimation schemes.

This paper is organized as follows. Section 2 provides the description of the general clock model. In Section 3, we review some existing clock synchronization protocols and describe several methods for estimating clock offset and skew in representative protocols such as RBS (Reference Broadcast Synchronization) [25] and TPSN (Timing-Sync Protocol for Sensor Networks) [29]. Lastly, Section 4 concludes this paper.

## 2. General Clock Model

**Figure 1.**
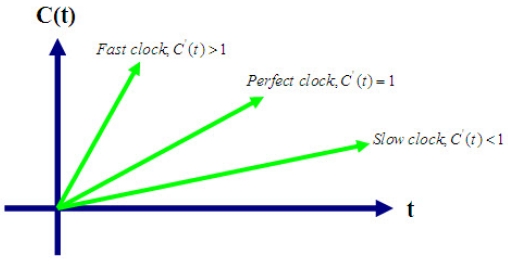
Behavior of fast, slow, and perfect clocks.

Computer clocks are, in general, based on crystal oscillators which provide a local time for each network node. The time in a computer clock is just a counter that gets incremented with crystal oscillators and is referred to as software clock. The interrupt handler must increment the software clock by one every time an interrupt occurs. Most hardware oscillators are not so precise because the frequency which makes time increase is never exactly right. Even a frequency deviation of only 0.001% would bring a clock error of about one second per day. Considering the physical clock synchronization in a distributed system to UTC (Universal Time Controller), the computer clock shows time C(t), which may or may not be the same as t, at any point of real time t. For a perfect clock, the derivative dC(t)/dt should be equal to 1. This term is referred to as clock skew. The clock skew can actually vary over time due to environmental conditions, such as humidity and temperature, but we assume that it stays bounded and close to 1, so that:

(1) $$1-\rho \leq \frac{dC(t)}{dt}\leq 1+\rho ,$$

where *ρ* denotes the maximum skew rate. A typical value of the maximum skew specified by the manufacturer for today's hardware is 10^-6^. We note that the clocks of two nodes are synchronized to one common time at some point of in time, but they do not stay synchronized in the future due to clock skew. Even if there is no skew, the clocks of different nodes may not be the same. Time differences caused by the lack of a common time origin are called clock phase offsets. Fig. 1 shows the behavior of fast, slow, and perfect clocks with respect to UTC [21, 22].

We next review some definitions related to clock terminology that have seen widely adapted in the literature. The time of a clock in a sensor node A is defined to be the function C_A_(t), where C_A_(t) = t for a perfect clock and the clock frequency is the rate at which a clock progresses. Clock offset is the difference between the times reported by the clocks at two sensor nodes; namely, the offset of clock C_A_ relative to C_B_ at time t is given by C_A_(t) – C_B_(t). Clock skew is defined to be the difference in the frequencies of two clocks (C′_A_(t) – C′_B_(t)), i.e., the rate of variation or derivative of clock offset. Another clock terminology is clock drift, which is the second derivative of the clock offset with regard to time. Mathematically, the drift of clock C_A_ relative to C_B_ at time t is (C′_A_(t) – C′_B_(t)) [21].

**Figure 2.**
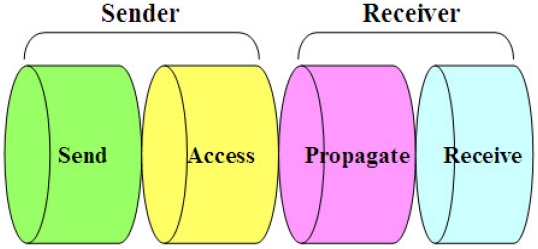
Decomposition of packet delay over a wireless channel.

In order for sensor nodes to be able to synchronize, they have to possess for a period of time a communication channel where the message delays between nodes can be reliably estimated. However, the enemy of accurate network time synchronization is the non-determinism of the delay estimation process. The estimation of latencies in channel is confounded by random events which bring about asymmetric round-trip-message delivery delays. This causes synchronization errors. The latency in channel can be decomposed into four distinct components [25]. Figure 2 illustrates the decomposition of packet delay when the packet travels over a wireless channel.

- Send Time: This is the time spent by the sender to construct the message, including kernel protocol processing and variable delays introduced by the operating system, e.g., context switches and system call overhead incurred by the synchronization application. This time also accounts for the time needed to transfer the message from the host to its network interface.
- Access Time: This is the delay incurred while waiting for access to the transmission channel. Access Time is very MAC (Medium Access Control)-specific. Contention-based MACs must wait for the channel to be clear before transmitting, and retransmit in case that a collision happened. Wireless RTS/CTS schemes such as those in 802.11 networks need an exchange of control packets before data transmission. TDMA channels require the sender to wait for its slot before transmitting.
- Propagation Time: This is the time for the message to travel from the sender to the destination node through the channel since it left the sender. In case that the sender and the receiver share access to the same physical media (e.g., neighbors in an ad-hoc wireless network or a LAN), this time is very small as it is simply the physical propagation time of the message through the medium. In contrast, Propagation Time dominates the delay in wide-area networks, where it includes queuing delay and switching delay at each router as the message transits through the network.
- Receive Time: This is the time for the network interface on the receiver side to get the message and notify the host of its arrival. This is typically the time required for the network interface to generate a message reception signal. If the arrival time is time-stamped at a low enough level in the host's operating system kernel (e.g., inside of the network driver's interrupt handler), Receive Time does not include the overhead of system calls, context switches, or even the transfer of the message from the network interface to the host and so can be kept small.

## 3. Clock Synchronization Protocols for Wireless Sensor Networks

Clock synchronization in wireless sensor networks has attracted a lot of attention in recent years. The development of p*ost-facto* synchronization by Elson and Estrin was a pioneering work [26]. In this method, unlike in conventional synchronization approaches such as NTP, local clocks of sensor nodes should normally operate unsynchronized at their own pace, but should synchronize whenever synchronization is needed. Local timestamps of two sensor nodes at the occurrence time of an event are synchronized later by extrapolating backwards to estimate the offset between clocks at a previous time. This synchronization method has laid down the ground for the RBS (Reference Broadcast Synchronization) protocol.

Since most networks are very closely associated with the application, therefore for the intended protocols used for synchronization are different from each other in some aspects and similar to one another in other aspects. In [21], the synchronization protocols are classified according to two kinds of features, which are synchronization related issues and application-dependent characteristics.

Synchronization issues
  - Master-slave versus peer-to-peer synchronization
    - Master-slave: This protocol assigns one node as the master and the other nodes as slaves. The slave nodes regard the local clock reading of the master node as the reference time and try to synchronize with the master. The representative examples in this class are the protocol of Mock et al. [41] and Ping's protocol [42].
    - Peer-to-peer: Any node can communicate directly with other nodes in the network. Such an approach removes the risk of the master node failure. Therefore these class of protocols are more flexible but also more uncontrollable. RBS [25] and the time diffusion protocol (TDP) [43] assume peer-to-peer configurations.
  - Internal synchronization versus external synchronization
    - Internal synchronization: A global time base is not available from within the system and therefore the protocol attempts to minimize the maximum difference between the readings of local clocks of the sensors. The protocol of Mock et al. [41] belongs to this scheme.
    - External synchronization: A standard time such as UTC (Universal Time Controller) is available and is used as a reference time. The local clocks of sensors seek to synchronize to this reference time. NTP [23] is the representative example.
  - Probabilistic versus deterministic synchronization
    - Probabilistic synchronization: This method gives a probabilistic guarantee on the maximum clock offset with a failure probability that can be bonded or determined. In a wireless environment where energy is scarce, this can be very expensive. The protocol of PalChaudhuri et al. [44] is a probabilistic variation of RBS [25].
    - Deterministic synchronization: Arvind [45] defined deterministic algorithms as those guaranteeing an upper bound on the clock offset with certainty. Most protocols are deterministic and so are RBS and TDP.
  - Sender-to-receiver versus receiver-to-receiver versus receiver-only synchronization
    - Sender-to-receiver synchronization (SRS): The sender node periodically sends a message with its local time as a timestamp to the receiver and then the receiver synchronizes with the sender using the timestamp received from the sender.
    - Receiver-to-receiver synchronization (RRS): This method uses the property that if any two receivers receive the same message in a single-hop transmission, they receive it at approximately the same time. Receivers exchange the time at which they received the same message and compute their offset based on the difference in reception times.
    - Receiver-only synchronization (ROS): A group of nodes can be simultaneously synchronized by only listening to the message exchanges of a pair of nodes.
  - Clock correction versus untethered clocks
    - Clock correction: The local clocks of nodes participating in the network are corrected either instantaneously or continually to keep the entire network synchronized. Timing-sync protocol for sensor networks (TPSN) [29] uses this approach.
    - Untethered clocks: Every node maintains its own clock as it is, and keeps a time-translation table relating its clock to the clock of the other nodes. Local timestamps are compared using the table. A global timescale is maintained in this way with the clocks untethered. RBS belongs to this approach.
  - Pairwise Synchronization versus network-wide synchronization
    - Pairwise synchronization: The protocols are primarily designed to synchronize two nodes, although they usually can be extended to deal with the synchronization of a group of nodes.
    - Network-wide synchronization: The protocols are mainly designed to synchronize a large number of nodes in the network.

Application-dependent features
  - Single-hop versus multi-hop networks
    - Single-hop communication: A sensor node can directly communicate and exchange messages with any other sensor in a single-hop network. The protocol of Mock et al. [41] is a representative example. However, it can be extended to multi-hop communication.
    - Multi-hop communication: Sensors in a domain communicate with sensors in another domain via an intermediate sensor relating to both domains. Communication can also occur as a sequence of hops through a chain of pairwise-adjacent sensors. RBS and TDP can be suitably extended to deal with multi-hop communication.
  - Stationary networks versus mobile networks
    - Stationary networks: Sensors do not move. The protocols such as RBS and TPSN, and Mock et al.'s protocol are geared to stationary networks.
    - Mobile networks: Sensors have the ability to move, and they connect with other sensors only when entering the geographical scope of those sensors. The changing topology is often a problem because it needs resynchronization of nodes and re-computation of the neighborhoods or clusters.
  - MAC-layer-based approach versus standard approach
    - RBS does not depend on MAC protocols so as to avoid a tight integration of the application with the MAC layer. On the other hand, the protocols proposed by Ganeriwal et al. [29] and Mock et al. [41] rely on the CSMA/CA protocol for the MAC layer.

Next we will summarize various synchronization protocols, discuss their advantages and disadvantages, and explain techniques for clock offset and skew estimation in several representative clock synchronization protocols. Given that sensor networks are generally closely related to the real world environment that they monitor, different networks present different characteristics impacting the synchronization requirements. For the rest of this section, we will describe the synchronization schemes explicitly designed and proposed for wireless sensor networks.

We will specifically consider the following protocols:

- Reference Broadcast Synchronization (RBS) [25]
- Timing-Sync Protocol for Sensor Networks (TPSN) [29]
- Delay Measurement Time Synchronization for Wireless Sensor Networks (DMTS) [42]
- Flooding Time Synchronization Protocol (FTSP) [46]
- Probabilistic clock synchronization service in sensor networks [44]
- Time Diffusion Synchronization Protocol (TDP) [43]

### 3.1. Reference Broadcast Synchronization [25]

**Figure 3.**
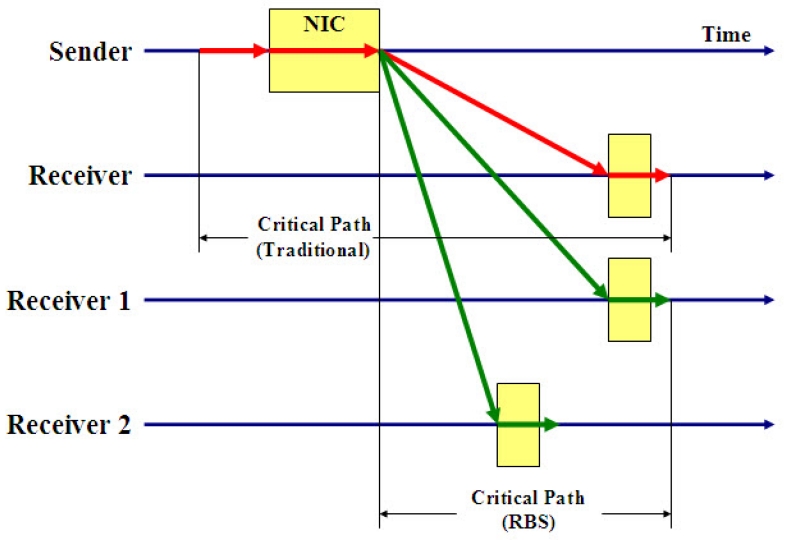
A critical path analysis for traditional time synchronization protocols and RBS.

Elson *et al.* proposed a synchronization protocol for sensor networks referred to as Reference Broadcast Synchronization (RBS) and that it is based on the *receiver-receiver synchronization*. The fundamental property of RBS is that a broadcast message is only used to synchronize a set of receivers with one another, in contrast with traditional protocols that synchronize the sender of a message with its receiver. By doing this, it removes the Send Time and Access Time from the critical path, as shown in Figure 3. This is a significant advantage for synchronization in a LAN, where the Send Time and Access Time are typically the biggest contributors to the non-determinism in the latency.

An RBS broadcast is always used as a relative time reference, and never to communicate an absolute time value. It is exactly this property that eliminates the error caused by the Send Time and Access Time: each receiver is synchronizing to a reference packet which was injected into the physical channel at the same instant. The message itself does not include a timestamp generated by the sender, nor is it important exactly when it is sent. As a matter of fact, the broadcast does not even need to be a dedicated time synchronization packet. Almost any extant broadcast can be used to recover timing information – for instance, ARP packets in Ethernet, or the broadcast control traffic in wireless networks (e.g., RTS/CTS exchanges or route discovery packets).

As mentioned above, RBS removes the effect of the error sources of Send Time and Access Time altogether; the two remaining factors are Propagation Time and Receive Time. The authors in [25] consider Propagation Time to be effectively 0. The propagation speed of electromagnetic signals through air is close to *c* (1*nsec*/foot), and through copper wire about 2*c*/3. For a LAN or ad-hoc network spanning tens of feet, propagation time is at most tens of nanoseconds, which does not contribute significantly to the *μ* sec-scale error budget. Moreover, RBS is only sensitive to the difference in propagation time between pair of receivers, as shown in Figure 3.

In order for a receiver node to interpret a message at all, it must be synchronized to the incoming message within one bit time. Latency caused by processing in the receiver electronics is irrelevant as long as it is deterministic, since the RBS scheme is only sensitive to differences in the receive time of messages within a set of receivers. Additionally, the system clock can easily be read at interrupt time when a packet arrives; this eliminates delays due to receiver protocol processing, context switches, and interface-to-host transfer form the critical path.

The simplest form of RBS is broadcasting a single pulse to two receiver nodes, enabling them to estimate their relative clock offsets. In other words, at first, a transmitter broadcasts a reference packet to two receivers (*i* and *j*) and then each receiver records the time when the reference packet was received, according to its own local clock. Finally, the receivers exchange their observation data. Based on this single broadcast alone, the receivers have sufficient information to form a local or relative timescale. This basic RBS scheme can be extended by allowing synchronization between *n* receivers by a single packet, where *n* may be larger than two or increasing the number of reference packets to obtain higher precision. Reference [25] shows via numeric simulation results that in the simplest case of two receivers, 30 reference broadcasts can improve the precision from 11 μsec to 1.6 μsec, after which there is a point of diminishing returns. The authors also make use of this redundancy for estimating clock skews. Instead of averaging the phase offsets from multiple observations, they performed a *least-squares linear regression*. This offers a fast, closed-form method for finding the best fit line through the phase error observations over time. The clock offset and skew of the local node with respect to the remote node can be recovered from the intercept and slope of the line.

Of course, fitting a line to observation data implicitly assumes that the frequency is stable, i.e., that the phase error is changing at a constant rate. The frequency of real oscillators changes over time due to environmental effects. In general, network time synchronization algorithms (for example, NTP) correct a clock's phase and its oscillator's frequency error, but do not try to model its frequency instability. In other words, frequency adjustments are made continuously based on a recent window of observations relating the local oscillator to a reference. The RBS system assumes also a similar scheme. RBS models oscillators as having high short-term frequency stability by ignoring data that is more than a few minutes old.

To test RBS, the authors implemented it on two different hardware platforms to assess its precision performance. The first used platform is the Berkeley Motes which is one of the most widely used sensor node architectures, and RBS acquired on this platform a synchronization precision within 11 μsec. The other platform is commodity hardware, Compaq IPAQs running Linux kernel v2.4, which is connected with an 11 Mbps 802.11 wireless network. The achieved precision of RBS on this platform was 6.29 ± 6.45 μsec.

The advantages of RBS are as follows [25]:

- The largest sources of nondeterministic latency can be eliminated from the critical path by using the broadcast channel to synchronize receivers with one another. This leads to significantly better precision synchronization than algorithms that measure round-trip delay.
- Multiple broadcasts enable tighter synchronization because residual errors tend to follow well-behaved distributions, and also allow estimation of clock skew and extrapolation of past phase offsets.
- Outliers and lost packets are handled gracefully; the best fit line can be drawn even if some points are missing.
- RBS allows nodes to construct local timescales. This is useful for sensor networks and other applications that require synchronized time but may not have an absolute time reference available.

On the contrary, RBS presents the following disadvantages [21, 25]:

- This protocol is not applicable to point-to-point networks; a broadcasting medium is needed.
- For a single-hop network of *n* nodes, RBS requires O(*n*^2^) message exchanges, which is computationally expensive in the case of large scale networks.
- Convergence time, which is the time taken to synchronize the network, can be high because of the large number of message exchanges.
- The reference node is left unsynchronized in this protocol. In some sensor networks, if the reference node needs to be synchronized, it will result in a considerable waste of energy.
  Now we take a look at a method for joint estimation of clock offset and skew in RBS.

#### 3.1.1 Estimation of clock offset and clock skew

**Figure 4.**
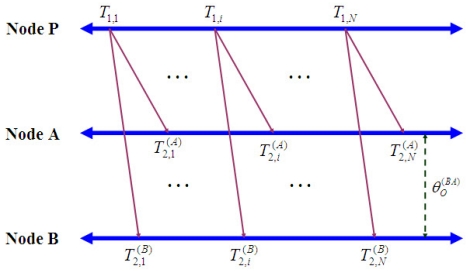
A clock synchronization model for the receiver-receiver synchronization approach (Node A and Node B).

As mentioned before, RBS is based on the *receiver-receiver synchronization* (RRS) scheme. RRS is an approach synchronizing a set of children nodes which receive the beacon messages from the parent node. Reference [27] suggested the maximum likelihood estimator of the relative clock offset which is equivalent to the estimator presented in [25]. The estimation of clock offset and skew in [27] is performed in accordance with the following approach. Consider a parent node P and arbitrary nodes A and B, which are located within the communication range of the parent node P, assuming, as illustrated in Figure 4, that both Node A and Node B receive the *i_th_* beacon from node P at time instants $T_{2,i}^{\left(A\right)}$ and $T_{2,i}^{\left(B\right)}$ of their local clocks, respectively. Nodes A and B record the arrival time of the broadcast packet according to their own timescales and then exchange their timestamps. Suppose that $X_{i}^{\left(PA\right)}$ denotes the nondeterministic delay components and *d*^(^*^PA^*^)^ represents the deterministic delay component from Node P to Node A. Then $T_{2,i}^{\left(A\right)}$ can be expressed as:

(2) $$T_{2,i}^{\left(A\right)}=T_{1,i}+d^{\left(PA\right)}+X_{i}^{\left(PA\right)}+\theta_{O}^{\left(PA\right)}+\theta_{S}^{\left(PA\right)}\cdot (T_{1,i}-T_{1,1}),$$

where *T*_1_,*_i_* is the transmission time at the reference node, $\theta_{O}^{\left(PA\right)}$ and $\theta_{S}^{\left(PA\right)}$ are the clock offset and skew of Node A with respect to the reference node, respectively. Likewise, at Node B:

(3) $$T_{2,i}^{\left(B\right)}=T_{1,i}+d^{\left(PB\right)}+X_{i}^{\left(PB\right)}+\theta_{O}^{\left(PB\right)}+\theta_{S}^{\left(PB\right)}\cdot (T_{1,i}-T_{1,1}).$$

Subtracting (3) from (2), we obtain the following equation:

(4) $$T_{2,i}^{\left(A\right)}-T_{2,i}^{\left(B\right)}=d^{\left(PA\right)}-d^{\left(PB\right)}+X_{i}^{\left(PA\right)}-X_{i}^{\left(PB\right)}+\theta_{O}^{\left(BA\right)}+\theta_{S}^{\left(BA\right)}\cdot (T_{1,i}-T_{1,1}),$$

where $\theta_{O}^{\left(BA\right)}=\theta_{O}^{\left(PA\right)}-\theta_{O}^{\left(PB\right)}$ and $\theta_{S}^{\left(BA\right)}=\theta_{S}^{\left(PA\right)}-\theta_{S}^{\left(PB\right)}$ are the relative clock offset and skew between Node A and Node B at the time they receive the *i^th^* broadcast packet from the reference node, respectively. Reference [27] assumes that the random delays, $X_{i}^{\left(PA\right)}$ and $X_{i}^{\left(PB\right)}$ are Gaussian random variables with mean *μ* and variance *σ*^2^/2.

Letting the noise component $z[i]=\mu^{\prime }+X_{i}^{\left(PA\right)}-X_{i}^{\left(PB\right)}$, where *μ*′ = *d*^(^*^PA^*^)^ − *d*^(^*^PB^*^)^ and *z*[*i*] ∼ *N*(*μ*′, *σ*^2^), $x[i]=T_{2,i}^{\left(A\right)}-T_{2,i}^{\left(B\right)}-\mu^{\prime }$, and *w*[*i*] = *z*[*i*] − *μ*′, we can write the set of observation data in matrix form as follows:

(5) x=Hθ+w,

where **x** = [*x*[1]*x*[2] ⋯ *x*[*N*]]*^T^*, **w** = [*w*[1]*w*[2] ⋯ *w*[*N*]]*^T^*, $\theta =[\theta_{O}^{\left(BA\right)}\theta_{S}^{\left(BA\right)}]$, and

(6) $$H={\left[\begin{array}{l} 11\cdots 1\\ 0T_{1,2}-T_{1,1}\cdots T_{1,N}-T_{1,1} \end{array}\right]}^{T}\cdot$$

By using standard results from estimation theory [28, Theorem 3.2, p. 44] and making some mathematical manipulations, the minimum variance unbiased (MVU) estimator for the relative clock offset and skew takes the expression [27]:

(7) $$\left[\begin{array}{l} \theta_{O}^{\left(BA\right)}\\ \theta_{S}^{\left(BA\right)} \end{array}\right]=\frac{1}{N\overset{N}{\underset{i=1}{\text{∑}}}D_{i}^{2}-{\left[\overset{N}{\underset{i=1}{\text{∑}}}D_{i}\right]}^{2}}\left[\begin{array}{l} \sum_{i=1}^{N}D_{i}^{2}\sum_{i=1}^{N}x[i]-\sum_{i=1}^{N}D_{i}\sum_{i=1}^{N}\left[D_{i}\cdot x[i]\right]\\ N\sum_{i=1}^{N}\left[D_{i}\cdot x[i]\right]-\sum_{i=1}^{N}D_{i}\sum_{i=1}^{N}x[i] \end{array}\right],$$

where *D_i_* = *T*_1_,*_i_* − *T*_1,1_. In case that there is no relative clock skew ( $\theta_{S}^{\left(BA\right)}=0$), the maximum likelihood estimator of the relative clock offset ${\hat{\theta }}_{O}^{\left(BA\right)}$ becomes

(8) $${\hat{\theta }}_{O}^{\left(BA\right)}=\frac{1}{N}\sum_{i=1}^{N}\left[T_{2,i}^{\left(A\right)}-T_{2,i}^{\left(B\right)}\right],$$

which is equivalent to the estimator presented in [25].

### 3.2. Timing-Sync Protocol for Sensor Networks [29]

**Figure 5.**
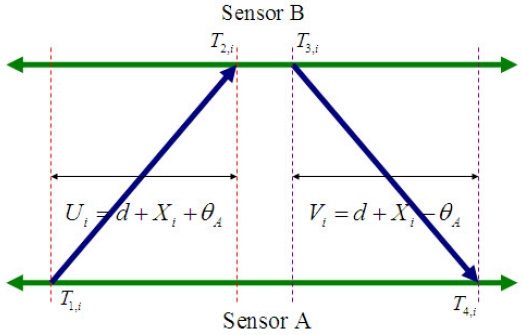
Two-way message exchange model with clock offset (*θ_A_* : clock offset, *d*: propagation delay, *X_i_, Y_i_* : random delays, *U_i_* = *T*_2_,*_i_* − *T*_1_,*_i_, V_i_* = *T*_4_,*_i_* − *T*_3_,*_i_*).

Ganeriwal *et al.* presented a network-wide clock synchronization protocol for sensor networks referred to as Timing-Synch Protocol for Sensor Networks (TPSN) [29], which relies on the traditional approach of sender-receiver synchronization. TPSN relies on the two-way message exchange scheme shown in Figure 5 to acquire the synchronization between two nodes. The authors argue that for sensor networks, the classical approach of implementing a handshake between a pair of nodes is better than synchronizing a set of receivers [30]. This observation comes as a result of time stamping the packets at the moment when they are sent, namely, at MAC layer, which is indeed feasible for sensor networks. The authors compared the performance of TPSN with that of RBS based on the receiver-receiver synchronization approach, and showed that TPSN provides about two times better performance, in terms of accuracy, than RBS on the Berkeley motes platform. They also illustrated that TPSN can synchronize a pair of motes to an average accuracy of less than 20 μsec and a worst-case accuracy of around 50 μsec.

The first step of the TPSN protocol is to create a hierarchical topology in the network. Each node is assigned a level in this hierarchical structure. A node belonging to level *i* can communicate with at least one node belonging to level *i - 1*. Only one node is assigned level 0, and it is referred to as the *root node*. This is done in the *level discovery phase*. Once the hierarchical tree structure is established, the root node initiates the second stage of the protocol, called the *synchronization phase*. In this second phase, a node with level *i* synchronize to a node with level *i – 1*. After all, every node is synchronized to the root node with level 0 and TPSN achieves network-wide time synchronization. Next we will describe the two phases in TPSN in some more detail.

#### Level Discovery Phase

This phase occurs at the deployment of the network. The root node is assigned a level 0 and it initiates this phase by broadcasting a *level_discovery* packet. The *level_discovery* packet includes the identity and the level of the sender node. The immediate neighbors of the root node receive this packet and assign themselves a level, one greater than the level of the packet they just received, i.e., the level *1*. After setting their own level, they broadcast a new *level_discovery* packet containing their own level. This procedure is continued and finally every node in the network is assigned a level. Once a node is assigned a level, it disregards any such future packets, which prevents flooding congestion from taking place in this phase. Therefore, a hierarchical structure is created with only the root node assigned to level 0. In general, a user node that acts as the gateway between the sensor network and the external world can act as the root node. The user node can be equipped with a GPS receiver, which enables the sensor nodes to be synchronized to the physical world.

#### Synchronization Phase

Pair wise synchronization is performed in this phase along the edges of the hierarchical structure constructed in the level discovery phase. As mentioned above, the classical approach of sender-receiver synchronization for implementing the handshake between a pair of nodes is used along each edge of the hierarchical tree. Consider a two-way message exchange between node A and node B as shown in Figure 5. At time *T*_1_,*_i_* (according to its local clock), node A sends a *synchronization_pulse* packet, which contains the level of node A and the value of *T*_1_,*_i_*, to node B. Node B receives this packet at time *T*_2_,*_i_* = *T*_1_,*_i_* + *d* + *θ_A_*, where *θ_A_* and *d* represents the clock offset between the two nodes A and B, and the propagation delay, respectively. Node B sends back an *acknowledgement* packet to node A at time *T*_3_,*_i_*. This packet carries the level of node B and the values of *T*_1_,*_i_, T*_2_,*_i_*, and *T*_3_,*_i_*. Then node A receives the acknowledgement packet at time *T*_4_,*_i_*. Assuming that the clock offset and the propagation delay is constant in this small span of time, node A can calculate the clock offset and propagation delay as illustrates by the equation (9) below, and synchronize itself to the clock of node B. This represents a sender-initiated approach, where the sender synchronizes its clock to that of the receiver:

(9) $$\theta_{A}=\frac{\left(T_{2,i}-T_{1,i})-(T_{4,i}-T_{3,i}\right)}{2};d=\frac{\left(T_{2,i}-T_{1,i})+(T_{4,i}-T_{3,i}\right)}{2}$$

This message exchange begins with the root node's initiating the synchronization phase by broadcasting a *time sync* packet. As soon as receiving this packet, nodes with level *1* wait for some random time before initiating the two-way message exchange with the root node, so as to avoid the contention in medium access. After receiving back an acknowledgement, these nodes adjust their clocks to the clock of the root node. The nodes with level *2* overhear this message exchange, and then they back off for some random time in order to ensure that the nodes with level *1* have completed their synchronization, after which they initiate the message exchange with nodes with level *1*. This process eventually enables all nodes to be synchronized to the root node.

The advantages of TPSN are as follows [21, 29]:

- It is scalable and the synchronization precision does not deteriorate significantly as the size of the network increases.
- Network-wide synchronization is computationally less expensive in comparison with such protocols as NTP [23].

On the other hand, TPSN has the following disadvantages [21, 29]:

- Energy conservation is not so effective since a physical clock correction needs to be performed on the local clocks of sensors while achieving synchronization.
- The protocol is not suitable for applications with highly mobile nodes because it requires a hierarchical infrastructure.
- TPSN does not support multi-hop communication.

Now let us take a look at some methods for estimating the clock offset and clock skew in a two-way message exchange model.

#### 3.2.1 Estimation of Clock Offset

Modeling of network delays in WSNs seems to be a challenging task [31]. Several probability distribution function (PDF) models for random queuing delays have been proposed so far, the most widely used being Gaussian, exponential, gamma, Weibull distributions [32, 33]. By the Central Limit Theorem (CLT), the PDF of the sum of a large number of independent and identically distributed (i.i.d.) approaches that of a Gaussian RV. This model is proper if the delays are thought to be the addition of numerous independent random processes. The Gaussian distribution for the clock offset errors was also reported by a few authors, such as [34], based on laboratory tests. On the other hand, a single-server M/M/1 queue can fittingly represent the cumulative link delay for point-to-point hypothetical reference connections, where the random delays are independently modeled as exponential random variables [35]. In this paper, we limit our presentation to mainly the situations where the portions of delays are Gaussian or exponential random variables.

Noh et al. proposed the maximum likelihood estimator (MLE) of clock offset in a two-way message exchange model, which will be deeply discussed below [36]. The authors suppose that the clock offsets of two nodes remain equal during the synchronization period, and the delays at *i*_th_ nodes *X_i_* and *Y_i_* are Gaussian random variables with mean *μ* and variance *σ*^2^/2. From Figure 5, *T*_2_,*_i_* and *T*_4_,*_i_* can be expressed as:

(10) $$T_{2,i}=T_{1,i}+d+\theta_{A}+X_{i},$$

(11) $$T_{4,i}=T_{3,i}+d-\theta_{A}+Y_{i},$$

where the variables *θ_A_, d, X_i_*, and *Y_i_* denote the clock offset between the two nodes, the propagation delay, and the variable portions of delays, respectively. After some mathematical manipulations, the likelihood function based on the observations ${\left\{U_{i}\right\}}_{i=1}^{N}$ and ${\left\{V_{i}\right\}}_{i=1}^{N}$ is given by:

(12) $$L(\theta_{A},\mu ,\sigma^{2})={\left(\pi \sigma^{2}\right)}^{-\frac{N}{2}}{\text{e}}^{-\frac{1}{\sigma^{2}}\left[\sum_{i=1}^{N}{\left(U_{i}-d-\theta_{A}-\mu \right)}^{2}+\sum_{i=1}^{N}{\left(V_{i}-d+\theta_{A}-\mu \right)}^{2}\right]},$$

where *N* stands for the number of message exchanges, *U_i_* = *T*_2_,*_i_* − *T*_1_,*_i_*, and *V_i_* = *T*_4_,*_i_* − *T*_3_,*_i_*. Differentiating the log-likelihood function gives:

(13) $$\frac{\partial \ln L(\theta_{A})}{\partial \theta_{A}}=-\frac{2}{\sigma^{2}}\sum_{i=1}^{N}\left[2\theta_{A}-(U_{i}-V_{i})\right].$$

Therefore, the MLE of clock offset is given as follows [36] under the assumption that there is no clock skew is given by [36]:

(14) $${\hat{\theta }}_{A}=\arg\underset{\theta_{A}}{\max}\left[\ln L(\theta_{A})\right]=\frac{\sum_{i=1}^{N}\left(U_{i}-V_{i}\right)}{2N}=\frac{\bar{U}-\bar{V}}{2}.$$

Thus, Node A can be synchronized to the node B by simply taking the difference of the average observations *Ū* and *V̄*. Noh et al. also proposed the joint MLE of clock offset and clock skew under the assumption of Gaussian random delays [36], a result which will not be detailed herein.

For exponential random delays *X_i_* and *Y_i_*, Jeske proved in [37] that the maximum likelihood estimator of clock offset, *θ_A_* exists when *d* is unknown and is the same form as the estimator proposed in [34], namely:

(15) $${\hat{\theta }}_{A}=\frac{\underset{1\leq i\leq N}{\min}U_{i}-\underset{1\leq i\leq N}{\min}V_{i}}{2}.$$

In case of one round of message exchange (*N* = 1), the MLE of clock offset for both Gaussian and exponential delay models is *θ̂_A_* (*U* − *V*)/2, which is exactly the same as the estimator presented in [29]. Notice further that the extension of MLE for joint estimation of clock phase offset and skew in networks with exponential delays was recently reported by Chaudhari *et al.* in [53].

In general, the delay distribution in the upstream, *F_X_* is not equal to that in the downstream *F_Y_*, because the node *A* → node *B* and node *B* → node *A* transmission paths through the network typically present different traffic characteristics, and thus the network delays in each path are potentially different. Equation (15) fits well the symmetric exponential delay model where both the uplink and downlink have the same exponential delay distributions. However, if this MLE is used in the asymmetric exponential delay model, there will be a bias in the clock offset. Therefore, it is necessary to achieve a more accurate estimate of the clock offset by using an alternate approach.

In [38], Lee *et al.* proposed a clock offset estimator using the bootstrap technique and bias correction method, which gives better performance than Jeske's MLE in the asymmetric exponential delay model. Specifically, bias-corrected estimators through non-parametric and parametric bootstrap were proposed. The procedures of bootstrap bias correction follow [39, 40]. These two bootstrap bias corrected estimators require Monte Carlo resampling of the empirical distribution functions. As far as the bootstrap bias correction method is concerned, in [54], Jeske had proposed a closed-form expression of the clock offset estimator by bootstrap bias correction approach based on the nonparametric technique and had compared analytically the estimator with Paxson's estimator [34] which was proved to be the MLE in [37]. Additionally, the effectiveness of bootstrap bias correction in the context of clock offset estimation was reported in [55] within the context of Pareto distribution, which was suggested in recent internet traffic modeling research.

At first, let us take a look at the clock offset estimation using bootstrap bias correction based on the nonparametric bootstrap method and the parametric bootstrap method, and then we will consider the estimation of the clock data offset using the particle filtering approach.

#### 3.2.2 Clock Offset Estimation Using Bootstrap Bias Correction

##### The Nonparametric Bootstrap

- **Step 0.** Conduct the experiment to obtain the random sample *X* = {*X*_1_, *X*_2_,… *X_n_*} and calculate the estimate *θ̂* from the sample *X*.
- **Step 1.** Construct the empirical distribution *F̂*, which puts equal mass 1/*n* at each observation *X*_1_ = *x*_1_, *X*_2_ = *x*_2_,… *X_n_* = *x_n_*.
- **Step 2.** From *F̂*, draw a sample $X^{*}=\left\{X_{1}^{*},X_{2}^{*},\ldots X_{n}^{*}\right\}$, called the bootstrap resample.
- **Step 3.** Approximate the distribution of *θ̂* by the distribution of θ̂* derived from the bootstrap resample X*.

##### The Parametric Bootstrap

Suppose that one has some partial information about *F*. For example, *F* is known to be the exponential distribution but with unknown mean *μ*. This suggests that we should draw a resample of size *n* from the exponential distribution with mean *μ̂*, where *μ̂* is estimated from *X* rather than from a non-parametric estimate *F̂* of *F*. We use the exponential distribution in the suggested bias correction approach through parametric bootstrapping. The parametric bootstrap principle is almost the same as the above non-parametric bootstrap principle, except some steps.

##### The Bootstrap Estimate of Bias

Let us suppose that an unknown probability distribution *F* has given the data x = (*x*_1_, *x*_2_,…*x_n_*) by random sampling, *F →* x. We want to estimate a real-valued parameter *θ* = *t*(*F*). For now we will assume the estimator to be any statistic *θ̂* = *s*(x). The *bias* of *θ̂* = *s*(x) as an estimate of *θ* is defined to be the difference between the expectation of *θ̂* and the value of the parameter *θ*:

(16) $$\text{bia}{\text{s}}_{F}=\text{bia}{\text{s}}_{F}(\hat{\theta },\theta )={\text{E}}_{F}\left[s(\text{x})\right]-t(F).$$

A large bias is usually an undesirable aspect of an estimator's performance. We can use the bootstrap to assess the bias of any estimator *θ̂* = *s*(x). The *bootstrap estimate of bias* is defined to be the estimate bias*_F̂_* obtained by substituting *F̂* for *F*:

(17) biasF^=EF^[s(x∗)]−t(F^).

For most statistics that arise in practice, the ideal bootstrap estimate bias*_F̂_* must be approximated by Monte Carlo simulations. We generate independent bootstrap samples x*^1^, x*^2^,⋯, x*^B^, evaluate the bootstrap replications θ̂*(*b*) = *s*(x*^b^), and approximate the bootstrap expectation E*_F̂_* [*s*(x*)] by the average:

(18) $$\hat{\theta^{*}}(•)=\sum_{b=1}^{B}{\hat{\theta }}^{*}(b)/B=\sum_{b=1}^{B}s({\text{x}}^{*b})/B.$$

The bootstrap estimate of bias based on the B replications bias^B, is (16) with θ̂*(●) substituted for E*_F̂_* [*s*(x*)]:

(19) bias^B=θ^∗(•)−t(F^).

###### ◆ Bias Correction

The usual reason why we want to estimate the bias of *θ̂* is to correct *θ̂* so that it becomes less biased. If bias^ is an estimate of bias*_F_* (*θ̂, θ*), then the obvious bias-corrected estimator is:

(20) θ¯=θ^−bias^.

Taking bias^ equal to bias^B=θ∗^(•)−θ^ gives:

(21) $$\bar{\theta }=2\hat{\theta }-\hat{\theta^{*}}(•).$$

**Figure 6.**
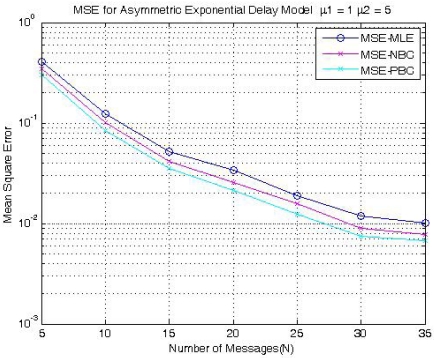
MSEs of clock offset estimators for asymmetric exponential delays (*μ*_1_ = 1, *μ*_2_ = 5).

Figure 6 shows simulation results comparing the mean squared error performance of Jeske's MLE of clock offset with those of clock offset estimators based on the bootstrap bias correction methodology described above in a two-way message exchange scheme under the assumption of asymmetric exponential random delays. The notations *μ*_1_ and *μ*_2_ denote the exponential delay parameters for the uplink and the downlink delay distributions, respectively. MSE-MLE, MSE MSENBC, and MSE-PBC denote the mean squared error (MSE) of Jeske's MLE, which is the MLE in the exponential delay model, the MSE of the bias-corrected estimator through nonparametric bootstrapping, and the MSE of the bias-corrected estimator through parametric bootstrapping, respectively. It is clear that the performances of the bias-corrected estimators are improved in an asymmetric exponential delay model and the bias corrected estimator through the parametric bootstrapping method has the best performance for the asymmetric exponential delay distributions.

#### 3.2.3 Clock Offset Estimation via Particle Filtering

In Figure 5, the *k_th_* up and down link delay observations corresponding to the *k_th_* timing message exchange are assumed to be given by *U_k_* = *T*_2_,*_k_* − *T*_1_,*_k_* = *d* + *θ_A_* + *X_k_* and *V_k_* = *T*_4_,*_k_* − *T*_3_,*_k_* = *d* + *θ_A_* + *Y_k_*, respectively. The fixed value *θ_A_* denotes the clock offset between the two nodes. *X_k_* and *Y_k_* denote the variable portions of *U_k_* = *T*_2_,*_k_* − *T*_1_,*_k_* = *d* + *θ_A_* + *X_k_* delays, which are assumed to be any distributions such as Gaussian, exponential, Gamma, and Weibull. Given the observation samples **z***_k_* = [*U_k_, V_k_*]*^T^*, the goal is to find minimum variance estimates of the unknown clock offset *θ_A_*. For convenience, we adopt the new notation *x_k_* = *θ_A_*. Thus, we are looking to determine:

(22) $${\hat{x}}_{k}=E\{x_{k}|Z^{K}\},$$

where **Z***^K^* denotes the set of observed samples up to time *K*, **Z***^K^* = {**z**_0_, **z**_1_,⋯,**z***_K_*}.

Since the clock offset value is constant, the clock offset is assumed to obey a Gauss-Markov dynamic state-space channel model [47] of the form:

(23) $$x_{k}=Fx_{k-1}+v_{k-1},$$

where *F* stands for the state transition matrix of clock offset. Since the clock offset is constant, we set *F* = 1. The noise vector *v_k_* is Gaussian random vector with zero mean and covariance $E[v_{k}^{}v_{k}^{T}]=Q$.

The vector observation model follows from the observed samples and assumes the expression:

(24) $$\begin{array}{l} z_{k}=\left[\begin{array}{l} d+x_{k}+X_{k}\\ d-x_{k}+Y_{k} \end{array}\right]=\left[\begin{array}{l} 1\\ 1 \end{array}\right]d+\left[\begin{array}{l} 1\\ -1 \end{array}\right]x_{k}+n_{k},\\ =Ad+Hx_{k}+n_{k} \end{array}$$

where **A** = [1,1]*^T^*, **H** = [1,−1]*^T^* and the observation noise vector **n***_k_* = [*X_k_, Y_k_*] might assume any distribution. In symmetric Gaussian delay models, **n***_k_* is a zero-mean Gaussian random vector with covariance **R** = σ^2^**I**. From the above discussion, the problem of estimating the clock offset can be formulated as a Gauss-Markov model with unknown states as depicted by the equations (23) and (24).

Under the Bayesian framework, an emergent technique for obtaining the posterior probability density function (PDF) is known as particle filtering (PF). PF is based on Monte Carlo simulations with sequential importance sampling (SIS). These methods allow for a complete representation of the posterior distribution of the states using sequential importance sampling and resampling [48] for the various probability densities. Since the true posterior PDF embodies all the available statistical information about the channel estimates, PF is optimal in the sense that all the available information has been used.

The posterior density *p*(*x*_0:_*_k_*÷**z**_1:_*_k_*), where *x*_0:_*_k_* = {*x*_0_, ⋯, *x_k_*} and **z**_1:_*_k_* = {**z**_1_, ⋯, **z***_k_*}, constitutes the complete solution to the sequential estimation problem. In many applications, such as tracking, it is of interest to estimate one of its marginals, namely the filtering density *p*(*x_k_*÷**z**_1:_*_k_*). By computing the filtering density recursively, we do not need to keep track of the complete history of the states. Therefore, from a storage point of view, the filtering density is more parsimonious than the full posterior density function. If we know the filtering density, we can easily derive various estimates of the system's states including means, modes, medians and confidence intervals. We show how the filtering density may be approximated using sequential importance sampling techniques.

**Figure 7.**
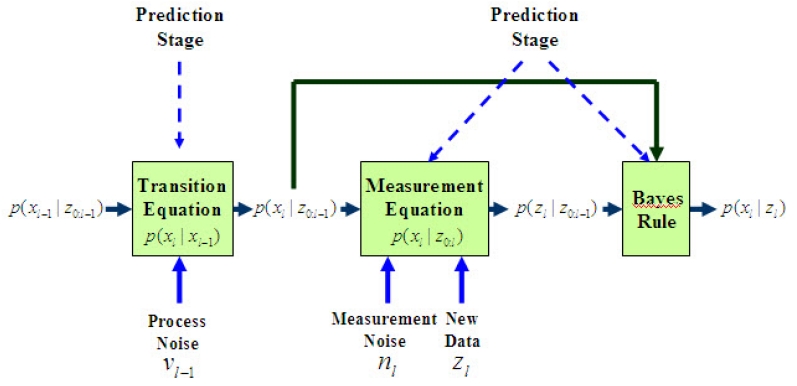
The recursive computation of the filtering density.

The filtering density is estimated recursively in two stages: prediction and update (correction), as illustrated in Figure 7. In the prediction step, the filtering density is propagated into the future via the transition density as follows,

(25) $$p(x_{k}|z_{0:k-1})=\int p(x_{k}|x_{k-1})p(x_{k-1}|z_{0:k-1})dx_{k-1}.$$

The transition density is defined in terms of the probabilistic model governing the states' evolution (23) and the process noise statistics.

The update stage involves the application of Bayes' rule when new data is observed [49, 50]

(26) $$p(x_{k}|z_{0:k})=\frac{p(z_{k}|x_{k})p(x_{k}|z_{0:k-1})}{p(z_{k}|z_{0:k-1})},$$

where the normalizing constant, *p*(**z***_k_*÷**z**_0_:*_k_*_−1_), depends on the likelihood function *p*(**z***_k_*÷*x_k_*) that is defined in terms of the measurements model (24), and *p*(*x_k_*÷**z**_0_:*_k_*_−1_) is the prior information. This is a central issue in Bayesian inference. In order to recursively evaluate the Bayesian equation (26), we utilize the Sequential Importance Sampling (SIS) algorithm. Again, the key idea is to represent the required posterior density function by a set of particles with associated weights and to compute estimates based on these samples and weights. As the number of particles becomes very large, this Monte Carlo characterization becomes an equivalent representation to the usual functional description of the posterior PDF, and the PF approaches the optimal Bayesian estimate. If we know the posterior density function, we can easily derive various estimates of the system's states including means, modes, medians and confidence intervals. Thus the posterior density at *k* can be approximated as:

(27) $$p(x_{0:k}|z_{1:k})\approx \sum_{i=1}^{N}w_{k}^{\left(i\right)}\delta (x_{0:k}-x_{0:k}^{\left(i\right)}),$$

where ${\left\{x_{0:k}^{\left(i\right)}\right\}}_{i=1}^{N}$ are a set of particles drawn from the posterior distribution and *δ*(●) is the Dirac delta function. The weights ( $w_{k}^{\left(i\right)}$) themselves can be shown to be updated as [50]:

(28) $$w_{k}^{\left(i\right)}=w_{k-1}^{\left(i\right)}\frac{p(z_{k}|x_{k}^{\left(i\right)})p(x_{k}^{\left(i\right)}|x_{k-1}^{\left(i\right)})}{q(x_{k}^{\left(i\right)}|x_{k-1}^{\left(i\right)},z_{k})},$$

where the proposal distribution $q(x_{k}^{\left(i\right)}|x_{k-1}^{\left(i\right)},z_{k})$ represents all the a priori knowledge. It is, however, usually difficult to sample directly from a given posterior distribution. Thus we choose what can be called a proposal distribution that is a probability distribution from which we can easily sample [48]. The selection of the proposal function is one of the most critical design issues in importance sampling algorithms and is the source of the main concern. The more accurate the proposal is to the true posterior, the better the performance of the particle filter is. It is often convenient to choose the proposal distribution to be the prior [51]:

(29) $$q(x_{k}|x_{k-1}^{\left(i\right)},z_{k})=p(x_{k}|x_{k-1}^{\left(i\right)}).$$

Again, we choose the stochastic model given by (23) as our model for the proposal distribution. As a result of not incorporating the most recent observations, this would seem to be the most common choice of proposal distribution since it is intuitive and can be implemented easily. This has the effect of simplifying (28) to:

(30) $$w_{k}^{\left(i\right)}=w_{k-1}^{\left(i\right)}p(z_{k}|x_{k}^{\left(i\right)}).$$

The update weights are based on the likelihood function. New estimates of the posterior are then computed based on the previous samples.

A common problem with the SIS particle filter is the degeneracy phenomenon, where after a few iterations, all but one particle will have negligible weights. It has been shown [52] that the variance of the importance weights can only increase over time, and thus it is impossible to avoid the degeneracy phenomenon. A large number of samples are thus effectively removed from the sample set because their importance weights become numerically insignificant. To avoid this degeneracy, a resampling stage may be used to eliminate samples with low importance weights and multiply samples with high importance weights. A common heuristic used to maintain an appropriate number of particles is to first calculate the effective sample size *N_eff_* introduced in [48, 51], and defined as:

(31) $${\hat{N}}_{eff}=1/(\sum_{i=1}^{N}{\left(w_{k}^{\left(i\right)}\right)}^{2}).$$

A threshold number of particles *N_th_* is then defined such that *N_th_* ≤ *N*. Multiple resamplings of particles then becomes necessary whenever *N_eff_* ≤ *N_th_*.

We have so far explained how to compute the importance weights sequentially and how to improve the sample set by resampling. The essential structure of the PF to clock offset estimation using the proposal function (29) can now be presented in terms of the following pseudo-code.

**Algorithm 1**. PF algorithm.Initialize weights (*k* = 0)  • Draw 
$x_{l}^{\left(i\right)}∼p(x_{0}),i=1:N$ Step.1) Prediction : predict via the state model (23)${\bar{x}}_{k+1}^{\left(i\right)}=Fx_{k}^{\left(i\right)}+v_{k}^{\left(i\right)},i=1:N$ Step.2) Measurement Update :  • Evaluate the weights according to the likelihood function as (30), *i* = 1:*N*$w_{k+1}^{\left(i\right)}=w_{k}^{\left(i\right)}p(z_{k+1}|{\bar{x}}_{k+1}^{\left(i\right)})$  • Normalize the weights 
${\hat{w}}_{k+1}^{\left(i\right)}=w_{k+1}^{\left(i\right)}/\sum_{j=1}^{N}w_{k+1}^{\left(j\right)}$ Step.3) Resampling Stage  • If *N_eff_* < *N_th_*   - take N samples with replacement from the set 
${\left(x_{k}^{\left(i\right)}\right)}_{i=1}^{N}$ where the probability to take sample *i* is 
$w_{k}^{\left(i\right)}$. Let 
$w_{k}^{\left(i\right)}=1/N$. Step.4) Output : MMSE${\bar{x}}_{k}=E[x_{k}|z_{k}]\approx \frac{1}{N}\sum_{i=1}^{N}x_{k}^{\left(i\right)}$ Step.5) Continue: set *k* → *k* + 1 and iterate to Step. 2.

Finally, we now introduce the PF with Bootstrap Sampling (BS) approach that integrates the PF with the BS for estimating the clock offset. The basic idea is quiet straightforward. In order to provide a large amount of observation data, we generate sampled observation data from the original observation data set by using the BS procedure. Then, we estimate the clock offset based on the PF. The important thing to check is how close the PDF of sampled data is to the true PDF. However, in case of less observation data, the performance's limitation is related to the finite number of observation data. Therefore, the solution is to overcome this limitation in the presence of reduced number of observation data. BS assumes additional data samples relative to the original data samples; these additional samples are defined by drawing at random with replacement. Each of the bootstrap samples is considered as new data. Based on the BS, we will increase the observation data set. Given a large number of new observation data, we can then approximate the clock offset by using PF. The following pseudo-code describes the procedure for estimating the clock offset via the nonparametric bootstrap sampling method.

**Algorithm 2**. PF with BS algorithm.Conduct the experiment to obtain the random sample **Z** = {**Z**_1_, ⋯ **Z***_K_*} and calculate the estimate *θ̂* from the sample **Z**.Step 1) Construct the empirical distribution ***H****^*, which puts equal mass 1/*n* at each observation {**Z**_1_ = **z**_1_, ⋯, **Z***_n_* = **z***_n_*}.Step 2) From ***H****^*, draw a sample 
$Z^{*}=\{Z_{1}^{*},\cdots ,Z_{1}^{*}\}$, called the bootstrap resample.Step 3) From the bootstrap resample Z*, estimate the clock offset *x̂* by PF.

**Figure 8.**
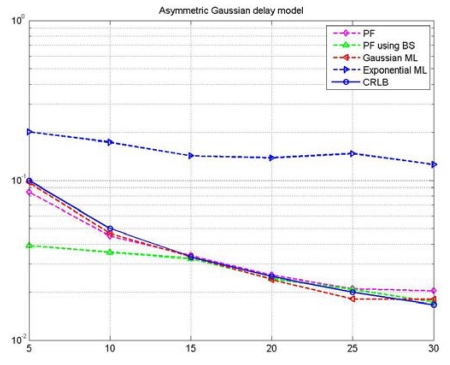
MSEs of clock offset estimators for asymmetric Gaussian random delay (σ = 1).

Figure 8 shows the MSE (Mean Squared Error) of several estimators and the Cramer-Rao Lower Bound (CRLB) assuming the random delay models are symmetric Gaussian delays. GML and EML denote the MSEs of Gaussian maximum likelihood and exponential maximum likelihood approaches, respectively. Note that the PF with BS performs much better with over 50% increase in performance when compared to the GML, EML, and even PF. Moreover, the PF with BS exhibits the best performance in the presence of small amount of data. As shown in Figure 8, it is notable that the performance limitation is related to the reduced number of observation data available. In the absence of measurement samples, BS offers the additional gain of pseudo measurement sampling in symmetric Gaussian delay models. There are 10 measurement samples, but from the bootstrap sampling, we can get a total of 20 measurement samples. Thus, due to the pseudo sampling samples, it turns out that the performance is improved. However, in the case of a large number of measurement samples, the gain becomes smaller. This means that the impacting factor on performance may be related to the bootstrap sampling error as well as to the number of measurement samples.

### 3.3. Delay Measurement Time Synchronization for Wireless Sensor Networks (DMTS) [42]

DMTS relies on a master-slave synchronization, sender-receiver synchronization, and clock-correction approach. This protocol was developed due to the need to develop a more suitable time synchronization method that avoids round trip time estimation. DMTS synchronizes the sender and multiple receivers at the same time and requires less number of message transfers than RBS. One of the characteristics of sensor networks is their self-organization and dynamic behavior. The self-organization feature implies that the network topology may change from time to time. DMTS focuses on scalability and flexibility, which means being either adaptive or insensitive to changes in network topology.

In this protocol, a leader is chosen as time master and broadcasts its time. All receivers measure the time delay and set their time as received master time plus measured time transfer delay. As a result, all sensors receiving the time synchronization message can be synchronized with the leader. The time synchronization precision is bounded mainly to how well the delay measurements are along the path.

**Figure 9.**
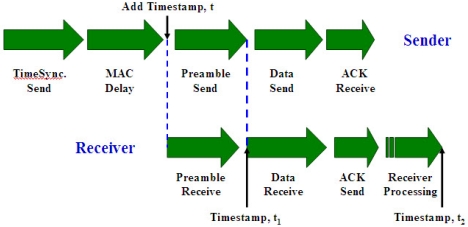
Time transfer path in a Mica mote.

Figure 9 shows the time line of transfer a time message from one node to another in Mica hardware platform. Assuming that the propagation delay is negligible, the total delay *t_d_* is measured as:

(32) $$t_{d}=t_{e}+(t_{2}-t_{1}),$$

where *t_e_* is the estimated time to transit the preamble and start symbols, *t*_1_ and *t*_2_ are receiver timestamps. Since a radio device has a fixed transmit rate, for instance, Mica radios transmit preamble and start symbols at the rate of 20 kbps, *t_e_* is a fixed delay and is expressed as *t_e_* = *nτ*, where *n* stands for the number of bits to transmit and *τ* denotes the time to transmit one bit over radio.

In the DMTS method, a time synchronization leader sends a time synchronization message with its timestamp *t*, which is added after MAC delay and a clear channel is detected. The receiver calculates the path delay and adjusts its local clock to *t_r_*:

(33) $$t_{r}=t+n\tau +(t_{2}-t_{1}).$$

The receiver is then synchronized with the leader. The lower bound of DMTS is the radio device synchronization precision, and the upper bound is the accuracy of local clock. Since DMTS needs only one-time signal transfer to synchronize all nodes within a single hop, it is energy efficient. It is also lightweight because there are no complex operations involved.

Multi-hop synchronization is also possible. If a node knows that it has children nodes, it broadcasts a time signal after it adjusts its own time. The node can now synchronize with its children by using single-hop time communication with a known-leader. To handle the situation when network nodes have no knowledge about their children, the concept of a time-source level is used to identify the network distance of a node from the master, which is selected by means of a leader selection algorithm. DMTS uses the concept of time source level to identify the distance from the master to another node. A time master assumes the time source level 0. A node synchronized with a level *n* receives a time source level *n* + 1. The root node broadcasts its time periodically and the synchronized nodes also do the same thing. On receiving a time signal, a node checks the time source level. If it is from a source of lower level than itself, it accepts the time; otherwise, it discards the signal. In this way, DMTS guarantees that the master time will be propagated to all network nodes with the number of broadcastings being equal to the number of the nodes. In addition, the algorithm warrants the shortest path to the time master, or the least number of hops, because a node always selects the node that is nearest to the time leader as its parent.

DMTS exhibits the following advantages [21, 42]:

- A user application interface is provided to monitor a wireless sensor network at run-time.
- Computational complexity is low and energy efficiency is quite high.

On the other hand, the disadvantages of the DMTS protocol are as follows [21, 42]:

- DMTS can be applied only to low resolution, low frequency external clocks.
- Synchronization precision is traded for the sake of low computational complexity and energy efficiency.

### 3.4. Flooding Time Synchronization Protocol (FTSP) [46]

The aim of the FTSP is to attain a network wide synchronization of the local clocks of participating nodes by using multi-hop synchronization. It is assumed that every node has a local clock exhibiting the typical timing errors of crystals and can communicate over an unreliable but error corrected wireless channel to its neighbor nodes. FTSP synchronizes the time of a sender to possibly multiple receivers making use of a single radio message time-stamped at both the sender and the receiver sides. MAC layer time-stamping can eliminate many of the errors, as shown in TPSN [29]. However, accurate clock-synchronization at discrete points in time is a partial solution only and thus compensation for the clock drift of the nodes is necessary for obtaining high precision in-between synchronization points and to keep the communication overhead low. Linear regression is used in this protocol to compensate for clock drift, which is already suggested in RBS [25].

As mentioned above, FTSP provides multi-hop synchronization. The root of the network – a single, dynamically elected node – keeps the global time and all other nodes synchronize their clocks to that of the root. The nodes form an ad-hoc structure to transfer the global time from the root to all the other nodes, as opposed to the fixed spanning-tree based approach proposed in [29]. This saves the initial phase of establishing the tree and is more robust against node and link failures, and changes in network topology [46].

### 3.5. Probabilistic Clock Synchronization [44]

This protocol is an extension of the deterministic RBS protocol for providing probabilistic clock synchronization. Arvind [45] defined a probabilistic clock synchronization protocol for wired networks. However, most synchronization protocols are based exclusively on deterministic algorithms. Deterministic methods have an advantage that they usually guarantee an upper bound on the error in clock offset estimation. However, in case that the system resources are badly constrained, a guarantee on synchronization accuracy may result in a large number of messages being exchanged during synchronization. In these cases, probabilistic algorithms can provide reasonable synchronization precision with lower computational and network overhead than deterministic protocols.

Elson *et al.* [25] found the distribution of the synchronization error among a set of receivers. Multiple messages are sent from the sender to the receivers. The difference in the actual reception times at the receivers is plotted. As each of these pulses are independently distributed, the difference in reception times yields a Gaussian distribution with zero mean.

Given a Gaussian probability distribution for the synchronization error, it is possible to calculate the relationship between a given maximum error in synchronization and the probability of actually synchronizing with an error less than the maximum error. If *e*_max_ is the maximum error allowed between two synchronizing nodes, then the probability of synchronizing with an error *e* ≤ *e*_max_ is given by:

(34) $$P(|e|\leq e_{\max})=\frac{\int_{-e_{\max}}^{e_{\max}}e^{-x^{2}/2}dx}{\sqrt{2\pi }}.$$

Therefore, as the *e*_max_ limit increases, the probability of failure (1−*P*(|*e*| ≤ *e*_max_)) decreases exponentially.

Based on equation (34), PalChaudhuri *et al.* [44] derived expressions converting the size of maximum clock synchronization error (service specifications) to the number of messages and the synchronization overhead (actual protocol parameter). The probability for the achieved error being less than the maximum specified error is given by:

(35) $$P(|e|)\leq e_{\max})=2\text{erf}\frac{\sqrt{n}e_{\max}}{\sigma }.$$

In equation (35)*n* stands for the minimum number of synchronization messages to guarantee the minimum allowed error and σ denotes the standard deviation of the distribution.

In [44], the relationship between the synchronization period and the maximum specified clock skew is also described. Given a maximum value for clock skew, a time period is derived within which re-synchronization must be done:

(36) $$\gamma_{\max}=e_{\max}+(T_{\text{sync}}+\sigma_{\max})\rho ,$$

where *γ*_max_ is the maximum allowable synchronization period at any point in time, *T_sync_* is the time period between synchronization points for the Always On model (time period of validity for Sensor Initiated model), ρ is the maximum drift of the clock rate, and σ_max_ is the maximum delay (after the synchronization procedure was started) in the time values of one receiver reaching another receiver [44].

This algorithm can be possibly extended to create a probabilistic clock synchronization service between receivers that may be multiple hops away from a sender. This extension is in contrast to the multi-hop extension used in RBS [25] assuming that all sensor nodes are always within a single hop of at least one sender. Moreover, the RBS algorithm requires the existence of a node which is within the broadcast region of both senders. This algorithm does not assume such assumptions, and sensor nodes herein are allowed to be multiple hops away from a sender and still be synchronized with all other nodes within the nodes transmission range of nodes.

The advantages of probabilistic clock synchronization service in sensor networks are as follows [21]:

- A probabilistic guarantee reduces both the number of messages exchanged among nodes and the computational load on each node.
- There is a tradeoff between synchronization accuracy and resource cost.
- This protocol supports multi-hop networks, which span several domains.

However, this method also presents disadvantages [21]:

- In case of safety-critical applications (for example, nuclear plant monitoring), a probabilistic guarantee on accuracy may not be proper.
- The protocol is sensitive to message losses. Nevertheless, it does not consider provisions for message losses.

### 3.6. Time Diffusion Synchronization Protocol [43]

TDP is a network-wide time synchronization protocol proposed by Su et al. [43]. Specifically, this protocol enables all the sensors in the network to have a local time that is within a small bounded time deviation from the network-wide equilibrium time. TDP architecture comprises many algorithms and procedures, which are used to autonomously synchronize the nodes, remove the false tickers (clocks deviating from those of the neighbors), and balance the load required for time synchronization among the sensor nodes. In the beginning, the sensor nodes may receive an *Initialize pulse* from the sink either through direct broadcast or multi-hop flooding. Then they determine for themselves to become master nodes with the *election/reelection of master/diffused leader node procedure (ERP)*, which is composed of the *false ticker isolation algorithm (FIA)* and *load distribution algorithm (LDA)*. At the end of the *ERP* procedure, the elected master nodes start the *peer evaluation procedure (PEP)* while other nodes do nothing. *PEP* helps to eliminate false tickers from becoming master nodes or diffused leader nodes.

After *PEP*, the elected master nodes start the *time diffusion procedure (TP)* through which they diffuse the timing information messages at every *δ* seconds for duration of *τ* seconds. Each neighbor node receiving these timing information messages self-determines to become a diffused leader node using the procedure *ERP*. Moreover, all neighbor nodes adjust their local clocks using the *time adjustment algorithm (TAA)* and the *clock discipline algorithm (CDA)* after waiting for *δ* seconds.

The elected diffused leader nodes diffuse the timing information messages to their neighboring nodes located within their broadcast range. This diffusion procedure allows all nodes to be autonomously synchronized. Additionally, the master nodes are re-elected at every *τ* seconds using the *ERP* procedure.

The following are the advantages of TDP [21]:

- This protocol is tolerant to message losses.
- A network-wide equilibrium time is achieved across all nodes and involves all the nodes in the synchronization process.
- The diffusion does not count on static level-by-level transmissions and thus it exhibits flexibility and fault-tolerance.
- The protocol is geared towards mobility.

On the other hand, the disadvantages are as follows.

- The convergence time tends to be high in case that no external precise time servers are used.
- Clocks may run backward. This can happen whenever a clock value is suddenly adjusted to a lower value.

## 4. Conclusions

Wireless sensor networks can be applied to a variety of applications and the common notion of time is necessary for a large number of sensor applications. This is due to the fact that the data from the sensors have to be collected and meaningfully fused to draw consistent inferences about the environment or the phenomenon being sensed. Some applications may operate on considerably precise time base, whereas other applications require energy efficiency by sacrificing the accuracy. It is very important to choose and apply the clock synchronization methods suitable to the purposes of the applications that WSNs aims at. In this paper, in order to satisfy this necessity, we described briefly the most representative clock synchronization protocols proposed for wireless sensor networks by reviewing their main characteristics.

This paper provides an in-depth analysis of the most representative protocols in wireless sensor networks, namely, RBS (Reference Broadcasting Synchronization) and TPSN (Timing-synch Protocol for Sensor Networks). In case of TPSN, we present not only the maximum likelihood estimate of clock offset, but also novel methods for estimating clock offset in WSNs which use nonparametric bootstrap and parametric bootstrap techniques, and particle filtering techniques. Due to these features, this review paper will help the researchers and designers in integrating diverse solutions to devise novel clock synchronization schemes that are best tailored for their specific applications.
